# A methodological evaluation of the published consensus statements, recommendations and guidelines about surgical management in the course of coronavirus disease pandemic

**DOI:** 10.1177/0218492320980937

**Published:** 2020-12-07

**Authors:** Luca Bertolaccini, Giorgio Lo Iacono, Antonio Mazzella, Elena Prisciandaro, Giulia Sedda, Lorenzo Spaggiari

**Affiliations:** 1 Division of Thoracic Surgery, IEO, European Institute of Oncology IRCCS, Milan, Italy; 2 Department of Oncology and Hemato-Oncology, University of Milan, Milan, Italy

**Keywords:** Delivery of health care, practice guidelines as topic, quality of health care, societies, medical

## Abstract

**Background:**

A project to benchmark the consensus statements, guidelines, and recommendations on surgical management in the course of the COVID-19 pandemic was developed to assess the methodology used. Standard and practical approaches for COVID-19 management in surgical patients to date are not accessible, despite the magnitude of the pandemic. A plethora of consensus statements, guidelines, and recommendations on surgical management in the course of COVID-19 epidemic have been rapidly published in the last three months.

**Methods:**

Each manuscript was scored on a seven-point scale in the different items and domains with the Appraisal of Guidelines for Research and Evaluation II.

**Results:**

Nine guidelines that met the inclusion criteria were assessed. Transnational cooperation produced only one guideline. Multivariable analysis showed that improved scores of stakeholders’ involvement were related to internationally developed guidelines. Clarity of presentation was related to the contribution of scientific societies due to greater rigor of development. The rigor of development produced guidelines with a high overall value. Higher healthcare expenses did not produce superior guidelines.

**Conclusions:**

Evaluated by the Appraisal of Guidelines for Research and Evaluation II, the methodological characteristic of consensus statements, guidelines, and recommendations on surgical management during COVID-19 pandemic was relatively low. International development should be recommended as a model for the development of best methodological quality guidelines.

## Introduction

Standard approaches for COVID-19 management in surgical patients do not presently exist, despite the extent of the epidemic and the parallels with previous coronavirus-associated diseases: Middle-East respiratory syndrome and severe acute respiratory syndrome.^1^ As COVID-19-dedicated resources expand, measuring the potential impact of COVID-19 has become a pertinent issue for national and global health programs. Wide-ranging interventional approaches have been suggested during the last three months. Nonetheless, quality studies on clinical trials are made challenging by the paucity of a standardized description of trial factors. Data coverage in trial depositories is particularly afflicted by internal inconsistencies, mainly for inclusion criteria or study endpoints.^1^ In the pandemic situation of COVID-19 and the consequent overload of intensive care units, surgeons had not only to reconsider the indications but additionally reduce the risk of COVID-19 infection in cancer patients. A plethora of current consensus statements, guidelines, and recommendations for the surgical organization during the COVID-19 pandemic have been published during the last three months. This paper aimed to assess the current literature to benchmark the methodological quality, with a focus on the analysis of variables that have affected the value of the research papers.

## Material and methods

The narrative literature search conducted up to 15 May 2020 is shown in Supplemental file 1. As previously described, Internet search engines and guideline databases were selected as proper.^2^,^3^ A mixture of keywords and subject headings were used to ensure a comprehensive search of the selected databases and websites. Independent searches were carried out for each concept and then merged using AND/OR. Subject headings and/or references mentioned in the results were verified where relevant. Irrelevant results and residual duplicates were manually removed. National recommendations were incorporated only if available in a peer-reviewed journal. The Appraisal of Guidelines for Research and Evaluation (AGREE) II instrument was used in the assessment of each guideline.^4^,^5^ AGREE II provides a structure to evaluate the quality of recommendations and guidelines, a methodological approach for the improvement of recommendations and guidelines, and direction on the best reporting approach. Each reviewer received a user’s manual of the AGREE II instrument, containing instructions. Table 1 shows the AGREE II items: 23 critical grouped within six domains and two overall ratings. Each item was rated on a 7-point scale (1 = strongly disagree, 7 = strongly agree) and captures a unique dimension of quality.^6^ As recommended in the AGREE II manual,^6^ a score was calculated for each domain: the higher the score, the better the methodological quality of the guideline in the corresponding AGREE II domain. The results for each recommendation or guideline were summarized for each domain. Additionally, the following data were recorded: nation, year of publication, language, affiliated scientific society, published in a peer-reviewed journal, and used at a local or international level. Financial records for the countries involved in the recommendations and guidelines were derived from the Organisation for Economic Cooperation and Development health statistics database.^7^ Organisation for Economic Cooperation and Development data contained: percentage of overall product assigned to health expenditure and the total sum of health expenses (per capita). Costs were switched to Euros as per the conversion rate on 23 May 2020.

**Table 1.** The evaluating domains in the Appraisal of Guidelines for Research and Evaluation (AGREE) II instrument. Each item has a 7-point scale.

Name of domain	Domains	No. of items	Description
Scope and purpose	D1	3	The overall aim of the guidelines, the specific health questions, and the target population
Stakeholder involvement	D2	3	The extent of development of the guideline related to the appropriate interested partiesThe views of its intended users
Rigor of development	D3	8	The process used to synthesize the evidence, the methods to formulate and to update the recommendations
Clarity of presentation	D4	3	Language, structure, and format of the guidelines
Applicability	D5	4	Possible barriers to implementation, strategies to improve uptake, and resource implications of applying the guidelines
Editorial independence	D6	2	Formulation of recommendations not biased with competing interests
Overall assessment		2	Overall quality and recommendation for the use of the guideline

As previously described, the characteristics of the recommendations and the AGREE II scores were studied descriptively.^2^,^3^ The Bravais-Pearson correlation coefficient measured the correlation between the AGREE II domains. For categorical factors, the analyses of variance were used. For continuous covariates, models assessed the AGREE II scores. Univariable analyses identified variables (*p* value < 0.30) for multivariable analyses. Logistic regression restricted the effect of confounding variables and identified the independent predictors that affected the scores. There was no adjustment for multiplicity because all analyses were exploratory. A *p* value < 0.05 was significant. Statistical analyses were made using RStudio (R version 3.5.3, Great Truth) with standard, ezr, rcmdr, and irr packages.^8^,^9^

## Results

Twenty-one guidelines were found in total. Four observers (GLI, AM, EP, GS) assessed nine guidelines closely fitting the inclusion criteria (Table 2).^10–18^ The evaluators found AGREE II simple and recognized it as helpful for refereeing the quality of the guidelines. A multinational collaboration produced only 1 guideline. Peer-reviewed journals published all guidelines. All evaluators rated all the AGREE II domains (no data missed). Table 3 shows the examination of the six domains scores related to the chosen guidelines. The lowest scores were received for the rigor of development domain (D3) in six (67.7%) guidelines. The guidelines from the French Society of Stomatology, Maxillofacial Surgery and Oral Surgery received the maximum score for the editorial independence domain (D6). The guidelines from the Canadian Society of Otolaryngology – Head & Neck Surgery and the French Society of Stomatology, Maxillofacial Surgery and Oral Surgery received the best overall assessments. The descriptive analysis of the domain and the overall assessment are shown in Table 4. The most significant scores were collected in the stakeholder involvement domain (D2). On the contrary, the rigor of development (D3) domains had the lowest possible score. The results of the univariable analyses of variance for the categorical variables are showed in Table 5. The involvement of a scientific society affected the scores of the rigor of development (D3) and stakeholder involvement (D2) domains. Transnationally, guidelines also had a slight impact on the editorial independence domain (D6). Lastly, the contribution of a scientific society also correlated with more clarity of presentation (D4) domains. The multivariable analysis (Table 6) showed that improved scores of stakeholders’ involvement (D2) and clarity of presentation (D4) were related to the involvement of a scientific society. Improved methodological quality with a high overall value was related to the better rigor of development (D3) domain. After the AGREE II appraisal, international development should be recommended as a model for the development of best methodological quality guidelines. A correlation was not detected between the covariate that calculates the economic status (Bravais-Pearson’s correlation coefficient = 0.10). Health spending as a percentage of the national overall domestic product was evaluated as a continuous variable. The total health expenses (per capita) were dichotomized matching to the median value (EUR 3,674.59). The countries with above-average spending on healthcare did not create significantly better recommendations (Table 7). The participation of scientific societies and the internationality similarly correlated with significant healthcare expenses. Quality was also mainly enhanced by recommendations-writing associations endorsing audits. Consequently, multivariate models were restricted to per capita health expenses. One domain, clarity of presentation (D4), affected the score in conjunction with a correction with health expenditure.

**Table 2.** Guidelines included in the Appraisal of Guidelines for Research and Evaluation (AGREE) II evaluation with information about the language and sources of retrieval.

Issuing societies	Titles of guidelines	Countries	Reference no.
National College of French Gynecologists and Obstetricians (CNGOF)	Recommendations for the surgical management of gynecological cancers during the COVID-19 pandemic – FRANCOGYN group for the CNGOF	France	10
Major Italian surgical and anesthesiologic societies	Surgery in COVID-19 patients: operational directives	Italy	11
Spanish Society of Otolaryngology and Head and Neck Surgery	Recommendations of the Spanish Society of Otolaryngology and Head and Neck Surgery for performing tracheotomies in patients infected by the coronavirus, Covid-19	Spain	12
Society of Thoracic Surgeons (STS)	Ramping up Delivery of Cardiac Surgery During the COVID-19 Pandemic: A Guidance Statement from The Society of Thoracic Surgeons COVID-19 Task Force	United States	13
Society of American Gastrointestinal and Endoscopic Surgeons (SAGES) and European Association for Endoscopic Surgery (EAES)	SAGES and EAES recommendations for minimally invasive surgery during COVID-19 pandemic	United States/European Union	14
French Association of Pediatric Otorhinolaryngology (AFOP) and French Society of Otorhinolaryngology (SFORL)	COVID-19 and ENT pediatric otolaryngology during the COVID-19 pandemic. Guidelines of the French Association of Pediatric Otorhinolaryngology (AFOP) and French Society of Otorhinolaryngology (SFORL)	France	15
French society of stomatology, maxillofacial surgery and oral surgery	Practitioners specialized in oral health and coronavirus disease 2019: professional guidelines from the French Society of Stomatology, Maxillofacial Surgery and Oral Surgery, to form a common front against the infectious risk	France	16
Canadian Society of Otolaryngology – Head & Neck Surgery (CSO-HNS)	Recommendations from the CSO-HNS taskforce on performance of tracheotomy during the COVID-19 pandemic	Canada	17
Thoracic Surgery Outcomes Research Network	COVID-19 guidance for triage of operations for thoracic malignancies: a consensus statement from Thoracic Surgery Outcomes Research Network	United States	18

**Table 3.** Appraisal of Guidelines for Research and Evaluation (AGREE) II scores by different domains of the analyzed guidelines.

Issuing society	Titles of guidelines	Scope and purpose (D1)	Stakeholder involvement (D2)	Rigor of development (D3)	Clarity of presentation (D4)	Applicability (D5)	Editorial independence (D6)	Overall assessment
National College of French Gynecologists and Obstetricians	Recommendations for the surgical management of gynecological cancers during the COVID-19 pandemic	50%	63%	26%	58%	48%	50%	42%
Major Italian surgical and anesthesiologic societies	Surgery in COVID-19 patients: operational directives	58%	67%	41%	33%	54%	46%	50%
Spanish Society of Otolaryngology and Head and Neck Surgery	Recommendations of the Spanish Society of Otolaryngology and Head and Neck Surgery for performing tracheotomies in patients infected by the coronavirus, Covid-19	57%	65%	32%	54%	38%	54%	42%
Society of Thoracic Surgeons	Ramping up delivery of cardiac surgery during the COVID-19 Pandemic: a guidance statement from the Society of Thoracic Surgeons COVID-19 Task Force	75%	46%	33%	67%	35%	41%	38%
Society of American Gastrointestinal and Endoscopic Surgeons (SAGES) and European Association for Endoscopic Surgery (EAES)	SAGES and EAES recommendations for minimally invasive surgery during COVID-19 pandemic	67%	47%	38%	63%	45%	69%	50%
French Association of Pediatric Otorhinolaryngology (AFOP) and French Society of Otorhinolaryngology (SFORL)	COVID-19 and ENT pediatric otolaryngology during the COVID-19 pandemic. Guidelines of the French Association of Pediatric Otorhinolaryngology (AFOP) and French Society of Otorhinolaryngology (SFORL)	52%	46%	51%	65%	53%	58%	50%
French Society of Stomatology, Maxillofacial Surgery and Oral Surgery	Practitioners specialized in oral health and coronavirus disease 2019: professional guidelines from the French Society of Stomatology, Maxillofacial Surgery and Oral Surgery, to form a common front against the infectious risk	56%	58%	27%	50%	48%	77%	25%
Canadian Society of Otolaryngology – Head & Neck Surgery (CSO-HNS)	Recommendations from the CSO-HNS taskforce on performance of tracheotomy during the COVID-19 pandemic	67%	60%	32%	54%	46%	59%	63%
Thoracic Surgery Outcomes Research Network	COVID-19 guidance for triage of operations for thoracic malignancies: a consensus statement from Thoracic Surgery Outcomes Research Network	67%	71%	63%	69%	59%	75%	63%

**Table 4.** Appraisal of Guidelines for Research and Evaluation (AGREE) II scores by domain with the inter-rater reliability between observers.

Domains	Mean	95%CI of the mean	Median	Minimum	Maximum
Scope and purpose (D1)	61.0%	54.59%–67.41%	58%	50%	75%
Stakeholder involvement (D2)	58.1%	50.73%–65.49%	60%	46%	71%
Rigor of development (D3)	38.1%	28.84%–47.39%	33%	26%	63%
Clarity of presentation (D4)	57.0%	48.46%–65.54%	58%	33%	69%
Applicability (D5)	47.3%	41.50%–53.16%	48%	35%	59%
Editorial independence (D6)	58.8%	49.06%–68.50%	58%	41%	77%
Overall assessment	47.0%	37.77%–56.23%	50%	25%	63%

CI: confidence interval.

**Table 5.** Analysis of variance for the six Appraisal of Guidelines for Research and Evaluation (AGREE) II domains.*

	Evaluated guidelines	AGREE II domains					
Factors		Scope and purpose (D1)	Stakeholder involvement (D2)	Rigor of development (D3)	Clarity of presentation (D4)	Applicability (D5)	Editorial independence (D6)
Level of the guidelines							
International	1 (11.1%)	67.00	47.0	38.0	63.0	45.0	69.0
National	8 (88.9%)	60.25	59.5	38.1	56.3	47.6	57.5
*p* value		0.482	0.243	0.993	0.601	0.768	0.118
Involvement of a scientific society				
Yes	8 (88.9%)	60.25	56.5	35.0	55.5	45.88	56.75
No	1 (11.1%)	67.0	71.0	63.0	69.0	59.0	75.0
*p* value		0.482	0.167	0.015	0.280	0.409	0.19
Overall assessment							
Adoptable	6 (66.7%)	62.5	55.8	42.17	57.83	49.0	61.0
Partially unadoptable	3 (33.3%)	58.0	62.67	30.0	55.33	44.0	54.33
*p* value		0.482	0.347	0.166	0.773	0.286	0.493

*Data are presented as *n* (%) or mean.

**Table 6.** Results of multivariable analysis.

Factor	AGREE II domains					
	Scope and purpose (D1)	Stakeholder involvement (D2)	Rigor of development (D3)	Clarity of presentation (D4)	Applicability (D5)	Editorial independence (D6)
Level of the guidelines (*p* value)		0.704	0.151			0.078
Involvement of a scientific society (*p* value)		0.008		0.086		0.674
Overall assessment (*p* value)		0.069	0.02		0.113	

**Table 7.** The impact of the economic situation on the Appraisal of Guidelines for Research and Evaluation (AGREE) II domains.

Factor	AGREE II domains					
	Scope and purpose (D1)	Stakeholder involvement (D2)	Rigor of development (D3)	Clarity of presentation (D4)	Applicability (D5)	Editorial independence (D6)
Percentage of gross domestic product dedicated to health expenditure (regression coefficient)	0.59	0.10	0.41	0.63	0.10	0.070
Health expenditure per capita (EUR)						
≤3,674.59 EUR	61.17	57.30	38.67	60.5	48.2	60.0
>3,674.59 EUR	60.67	59.67	37.0	50.0	45.7	56.3
*p* value	0.939	0.755	0.84	0.199	0.672	0.710

## Discussion

During the early stages of the pandemic, prioritization and deferral of non-urgent surgical patients had the scope to preserve personal protective equipment supplies, critical care resources, hospital beds, blood products, and maintain adequate healthcare workers for the pandemic flood. Besides, hospital boards recommended the delay in elective surgeries to protect healthcare personnel and patients by limiting exposure to asymptomatic carriers of the virus and COVID-19 patients. Nonetheless, the lower survival related to delaying surgical procedures needs hospitals to start planning for the resumption of surgery.^13^

Several accepted techniques for quality improvement in healthcare have been described. AGREE II was chosen based on previous reports.^2^,^3^ AGREE II should be used by healthcare workers who want to evaluate a guideline before adopting it into practice; by recommendations developers for a rigorous and structured methodology, to evaluate the guidelines, or to appraise recommendations from other groups for possible reworking; by policymakers to choose which guidelines to recommend, and by educators to improve critical appraisal skills teaching, core abilities in recommendations development, and reporting. AGREE-II is a tool designed to assist in the development, writing, and evaluation of practise guidelines and health system supervision, specifically to address variability in the quality of guidelines. Many of the so-called guidelines for the management of surgical patients during the COVID-19 pandemic are not necessarily meant to be evidence-based. They are much more fairly characterized as time-sensitive statements/recommendations intended to offer some needed guidance in times of uncertainty. This analysis of the standards of the surgical management of COVID-19 patients is up to date, and to the authors’ knowledge, the broadest systematic assessment. The nine guidelines assessed varied broadly in goals and content but had some similarities in the structure and subject.

Surgeons are obligated to practise evidence-based medicine, based on the interpretation of scientific reports on therapeutic advances. In this period of the COVID-19 pandemic, healthy scepticism should be retained. The principle of clinical balance, especially contemplating harmful interventions, should be employed. Otherwise, in the effort to cure patients now and efficiently, surgeons may fall victim to therapeutic errors and cognitive biases. Under circumstances of uncertainty-related anxiety and communication overload, the availability bias increased the inappropriate propensity to approve newly acquired information due to the ease of recall. Nevertheless, as preventive measures, we had to perform invasive procedures and surgeries. Thus far in the Covid-19 pandemic, surgical management has often been started and modified based on single case reports and surgeon’s editorials, instead of randomized trials. Therefore, surgeons should be working with clinical equipoise. An unprecedented biopsychosocial crisis characterizes these times. Furthermore, surgeons should be led by the voice of reason, analytically appraising evidence in deciding on the treatment of patients, and make use of anecdotal opinions just to produce assumptions for trials.^19^

This paper suffers from some limitations. The exclusion of studies published outside peer-reviewed journals was the main limitation of the narrative literature review. There was the risk of missing not usually indexed potential recommendations (e.g., documents from authoritative bodies and governments). Although reviewers with content-specific knowledge assessed the guidelines and recommendations, the use of AGREE II needs prudent interpretation because it is subjective. AGREE II is an instrument for methodological rigor and transparency of guideline development. Although subjective, AGREE II represents the gold standard. Nevertheless, there is no capacity for discernment from high- to low-quality benchmarks, so a low score domain may not always reproduce low quality. Furthermore, some guidelines did not report comprehensive methodology, so a low-score domain may not always reflect minimal quality. AGREE II could also be used to evaluate non-official documents published in scientific journals, or other recommendations that do not follow traditional development methodology. Consequently, the AGREE II overall scores should be clarified in specific contexts and with caution. There was a small chance that inaccurate estimations would be due to poor description because AGREE II requires an evaluation based on the descriptions published in the manuscripts. Consequently, the overall scores should be inferred in the right contexts and carefully.^4–6^ The AGREE II instrument can evaluate the discrepancies in substantial characteristics of clinical guidelines, and it can be utilized by a broad range of researchers in various scientific fields.

Guidelines, consensus statements, and recommendations accurately planned and described are essential in clinical decision-making. Even though the rigor of development was adequately reported, the overall methodological quality of consensus statements, guidelines, and recommendations on surgical management during the COVID-19 pandemic was judged moderately satisfactory. International development should be recommended as a model for the development of best methodological quality guidelines. Subsequently, it will be feasible for the development of the best methodological guidelines that will certainly manage clinical practice, beginning with a systematic review of the argument and following a rigorous methodology of development.
